# Dr. J. B. Hamilton and “One Water Wyman”

**Published:** 1897-05

**Authors:** 


Dr. J. B. Hamilton and "One Walter Wyman."—The truth of the saying that "Republics are ungrateful," finds a striking verification in the outrageous treatment that Dr. Hamilton has received at the hands of the United States Government. I know but one parallel in history,—and barring the republic part of it,—it is strikingly analogous; the sending home in chains of Christopher Columbus, after the benefits he had bestowed upon his country.

We are in receipt of a pamphlet entitled "*Letters and Telegrams and Documents Relating to the Transfer of Surgeon Hamilton.*" As the facts in the case are fully set forth in Dr. Hamilton's affidavit, we reproduce it here:

AFFIDAVIT IN SUPPORT OF PROTEST.

State of Illinois, }<br>County of Cook, } ss.

Personally appeared before me, a notary public in and for the said County of Cook and State of Illinois, John B. Hamilton, who on his oath doth depose and say that he is at present a Surgeon of the Marine Hospital Service of the United States; that he was originally appointed into said service in the year A. D. 1876, after a competitive examination. That in the year A. D. 1879 he was promoted to be Supervising Surgeon-General of the said service, being appointed thereto by the president of the United States, by and with the consent of the Senate of the United States, and that he held the said office and exercised the functions thereof according to law, and the direction of the Secretary of the Treasury for the time being, from the date of said appointment down to May 31, 1891, or thereabout. That during the month of May, 1891, one Walter Wyman, then a Surgeon in the Marine Hospital Service, was on duty in the Marine Hospital Bureau in the office of said Supervising Surgeon-General Hamilton, in the City of Washington, being duly detailed thereto by order of said Hamilton; and the said Wyman's duties were such as pertained to one of the medical assistants in the said office, and he, the said Wyman, was in fact one of the assistants in the office of the said Supervising Surgeon-General Hamilton. And the affiant doth further say that on or about the twelfth day of May he, the said Hamilton, was offered a professorship in the Rush Medical College of Chicago, being the chair of the principles of surgery and clinical surgery in the said college. That during the time the said offer was pending the said Wyman repeatedly and urgently asked him, the said Hamilton, to accept the offer of the said Rush Medical College, to relinquish the office as above-named, and to recommend his, the said Wyman's, appointment to the vacancy thus created. This personal importunity was followed by requests of friends of the said Wyman to the said Hamilton, urgently praying the promotion of the said Wyman, alleging as one of the principal reasons, the strong friendship of the said Wyman for him, the said Hamilton.

The affiant further says that the said Wyman assured him repeatedly that if he were appointed to the office that he would always have a friend therein; that he, the said Hamilton, would be detailed to have charge of the exhibit of the United States Marine Hospital Service at the World's Fair in Chicago, when opened, that when re-appointed surgeon, he could remain at the Chicago station so long as he, the said Hamilton, might desire, and in no event would he, the said Hamilton, be transferred to any other station without his consent. He, the said Wyman, renewed these statements to various persons, among them the Honorable Charles Foster, Secretary of the treasury, and to Mary L. Hamilton, the wife of the said John B. Hamilton. Inquiry was made by said Hamilton of the said Secretary of the Treasury, and of the President of the United States, then Gen. Benj. Harrison, whether in the event of the resignation of the said Hamilton as Surgeon-General and his re-appointment as surgeon he could be allowed to remain at the Chicago station two or more terms of four years each. Before consenting to this arrangement, the President of the United States, the said Benjamin Harrison, required him, the said Supervising Surgeon-General Hamilton, to submit to him names of medical officers of the Army, Navy and Marine Hospital Service who had held or were holding professorships in medical colleges. The said Hamilton in due time submitted to the President a list of the names of the officers as aforesaid who had in the past held, or were then holding professorships. The said President then consented to re-appoint the said Hamilton as surgeon and to appoint the said Wyman as Supervising Surgeon-General, on the conditions and with the understanding as aforesaid.

The affiant further says, that he came to the Chicago Station of the Marine-Hospital Service and has served there as surgeon and faithfully performed the duties of the said office from the day on or about the 15th day of June, A. D. 1891, up to and including the present time.

The affiant, on his oath, further says, that he has in good faith expected to be allowed to remain in charge of the Marine-Hospital Service for two or more terms, as allowed by paragraph 41 of the regulations governing the said service, whereby an officer may remain at any one port more than four years when specially authorized by the department, and that he in good faith considers that such special authorization has been given, by the consent of the former said Secretary of the Treasury and the President.

The affiant further states, that he has made arrangements with the said medical college and with the Trustees of the American Medical Association, based on the understanding as aforesaid, and he cannot, at this time, leave the said station without great hardship and pecuniary loss, and that the said Supervising Surgeon-General Walter Wyman, in violation of his agreement and understanding as aforesaid, has issued and caused to be mailed to him, the said Surgeon Hamilton, an order whereby he is directed to proceed to the port of San Francisco, California, and from the operations of that order he has respectfully appealed to the Secretary of the Treasury, for the reasons stated in this affidavit and in said appeal.

JOHN B. HAMILTON.

Sworn and subscribed to before me, a notary public in and for the county of Cook and State of Illinois, this the 18th day of September, A. D. 1896.

[Signed] ALTHEA B. OGDEN, Notary Public.

Dr. Hamilton, of course, protested against such injustice and appealed to his government,—to the Secretary of the Treasury and the great Surgeon-General, to reconsider it. The appeal was in vain. Leading members of the profession everywhere, congressmen, senators, and cabinet officers, joined in the appeal. The New York State Medical Association protested against the injustice of the act, and passed most complimentary resolutions, setting forth Dr. Hamilton's brilliant services to his country, and his claim to consideration; all in vain.

For some reason, unknown to us, Dr. Hamilton had fallen under the ban of the mighty man's displeasure, and must be gotten out of the way. He had used Hamilton as a stepping stone to power, and now must needs sacrifice him,—drive him out of the service. When reminded that Hamilton had made him Surgeon-General, and reminded him of his pledge, Wyman had the cool effrontery to reply that he "had no knowledge of any such agreement." When this statement is contrasted with the sworn statement of Doctor and Mrs. Hamilton, and Hon. Chas. S. Foster, Dr. Wyman is placed in a most unenviable light. In vain was the Secretary of the Treasury, Mr. Carlisle, appealed to. He hadn't the manhood to protect Dr.

Hamilton from the injustice of the petty despot's wrath,—the coward.

No man with any sense of justice or right can read the affidavit of Dr. Hamilton without indignation and disgust; a sense of shame that one filling the highest medical office of the government should be capable of such unmanly conduct. The culmination of the outrage was only reached later. When the time had nearly arrived when Hamilton should go to San Francisco, he was taken dangerously ill, and communicating the facts to his chief, asked for sick leave; and the miserable creature refused it!

Well, the Surgeon-General has driven the most useful and brilliant officer out of the government service. But he is not crushed. His greatness, by contrast with Wyman's pusillanimity, makes him a veritable giant. His deeds will live in the memory of an appreciative profession, and his great personal worth and professional ability will shine resplendent when the sanitary history of America is written, while the creature whom he, in the goodness of his heart, made the mistake of befriending, will be forgotten; or, if remembered at all, remembered as another Uriah Heep—a prototype of the ingrate!

Should the revolutionists carry their point, and secure the abolition of the Texas quarantine, surrender it to the United States government, *this* is the man who would have charge of it.

